# The Transition From Localized to Metastatic: A Case Report of Adult TFE3-Positive Xp11.2 Translocation Renal Cell Carcinoma

**DOI:** 10.7759/cureus.43378

**Published:** 2023-08-12

**Authors:** Mohammad Saad Naviwala, Tasneem Dawood, Zeeshan Uddin, Qurratulain Chundriger, Imran K Jalbani

**Affiliations:** 1 Oncology, Aga Khan University Hospital, Karachi, PAK; 2 Pathology and Laboratory Medicine, Aga Khan University Hospital, Karachi, PAK; 3 Urology, Aga Khan University Hospital, Karachi, PAK

**Keywords:** tfe3 gene fusion, surgical case report, radical nephrectomy, tyrosine kinase inhibitors, adult renal cell carcinomas (rcc) associated with xp11.2 translocations

## Abstract

Xp11.2 translocation renal cell carcinoma (Xp11.2 RCC) is a rare tumor, occurring more frequently in childhood than in adulthood. It results from Xp11.2 chromosome translocations and the fusion of the transcription factor E3 (TFE3) gene. In this context, we present a case report of an 18-year-old female who was diagnosed with Xp11.2 RCC following open radical nephrectomy and lymph node dissection on the left side. The histopathological analysis indicated stage T3aN1Mx disease, which was confirmed through immunohistochemistry (IHC) and fluorescent in situ hybridization (FISH). The patient remained under observation until March 2023 when systemic scans uncovered the presence of ascites, peritoneal carcinomatosis, and left supraclavicular lymphadenopathy. A subsequent biopsy reaffirmed the primary disease, leading to the planning of systemic treatment involving tyrosine kinase inhibitors (TKIs) and immunotherapy. However, due to financial constraints, the patient's treatment options were limited to sunitinib initially. The current plan involves reevaluation after three months using scans to determine the subsequent course of treatment. Our case report offers crucial insights into the clinical presentation, diagnosis, and treatment of this rare malignancy. This enhances medical understanding, guides research, and improves the management of similar cases. Case reports like this share practical experiences, shaping future studies and patient care.

## Introduction

Renal cell carcinoma (RCC) is a diverse malignancy, comprising various histological subtypes, including clear cell, papillary, chromophobe, and collecting duct carcinoma. Among these, Xp11.2 translocation RCC (Xp11.2 RCC) is a rare and genetically distinct disease entity, recognized by WHO's renal tumor classification scheme in 2004. This subtype involves the fusion of the transcription factor binding to the immunoglobulin heavy constant mu (IGHM) enhancer 3 (TFE3) on chromosome Xp11.2 with various partners [1]. The latest WHO classification in 2022 refers to this entity as TFE3-rearranged RCC [2].

Initially considered more common in pediatric and young adult populations, recent studies have revealed a higher prevalence of Xp11.2 RCC in adults, accounting for 0.95% to 5% of all adult RCCs [3]. Diagnosing Xp11.2 RCC relies on immunohistochemical studies and fluorescent in situ hybridization (FISH) rather than histological characteristics and imaging examinations, as it may morphologically resemble other RCC variants [4].

In adults, Xp11.2 RCC often presents at an advanced stage with distant metastasis, owing to its indolent nature [5]. Despite aggressive surgical interventions, the prognosis is generally poor, with patients experiencing rapid deterioration [5]. However, cases detected before distant metastasis with radical nephrectomy and lymph node dissection may have more favorable outcomes [6]. Unfortunately, some patients who undergo curative intent treatment may experience metastasis due to the lack of data in the adjuvant setting, as was the case with the young woman presented in this report [7].

Currently, no standardized surveillance protocol exists, making follow-up challenging for patients who undergo radical nephrectomy. Clinicians often consider Xp11.2 RCC a high-risk variant and emphasize its rarity and diverse presentations for appropriate patient management and surveillance [7].

In this report, we present a rare case of Xp11.2 RCC with TFE3 gene fusion in a young woman who had undergone curative intent treatment but, unfortunately, experienced metastasis due to the lack of data in the adjuvant setting.

## Case presentation

In April 2022, an 18-year-old unmarried female with no known comorbidities presented to the emergency room. She complained of unintentional weight loss (5 kg) over the past few months, left upper abdominal pain radiating to the flank region after a football injury four weeks ago, and fever for the last three days. A left lumbar lesion (17.3 x 11.6 cm) adjacent to the pancreatic tail and left lobe of the liver was detected on a prior abdominal ultrasound.

**Figure 1:**
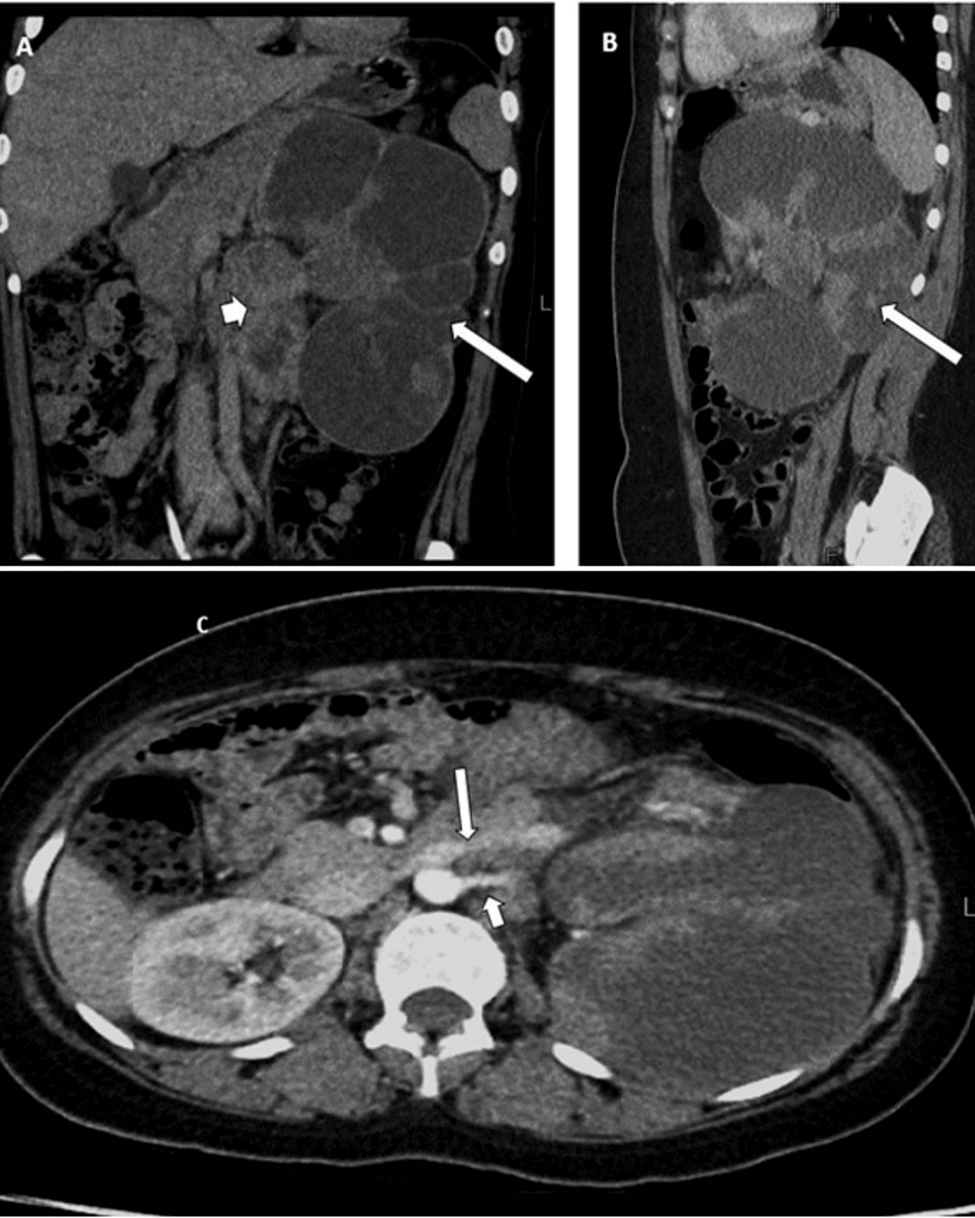
An 18-year-old female presented with left flank pain and weight loss for one month (A, B) Post-contrast CT scans revealed a large reniform multiloculated cystic lesion with solid-enhancing components, completely replacing the entire left kidney (long arrow). Additionally, conglomerate partly necrotic paraaortic lymph nodes were observed (short arrow). (C) An axial image showed the left renal artery (short arrow) and vein (long arrow) encased by the lymph nodal mass.

A contrast-enhanced CT scan revealed a large, complex, multiloculated cystic lesion in the left kidney, which replaced normal tissue. This lesion contains internal enhancing septae and multiple solid nodular components. Its size measures 184 x 104 mm, with a solid portion of 20 x 15 mm displaying a mean Hounsfield unit of 80 (Figure 1A). The lesion abuts the descending colon, left lateral chest wall without infiltration, and pushes against the tail of the pancreas and lower spleen border. It also contacts the posterior chest wall and left psoas muscle, and anteriorly abuts the transverse colon. Additionally, numerous enlarged retroperitoneal lymph nodes were identified, with the largest one measuring 35 x 43 mm in the left paraaortic region (Figure 1B). These lymph nodes encased the renal artery and vein; however, no tumor infiltration within the vessels was observed (Figure 1C). Radiologically, the tumor was staged as T3aN1Mx, corresponding to stage IIIA according to the 8th American Joint Committee on Cancer Classification.

The patient's bone scan was negative for bone metastasis. Blood tests indicated microcytic anemia with a hemoglobin level of 5.8 mg/dL, hematocrit of 21.3%, and mean corpuscular volume of 69 fl. Other lab results were normal, including creatinine at 0.9 mg/dL. The patient had Eastern Cooperative Oncology Group status 1 and no history of alcohol, smoking, or drug abuse. The patient had no family history of malignancy. Before surgery, two packs of red blood cells were transfused to address the anemia.

Before the procedure, a CT-guided biopsy of the left paraaortic lymph node confirmed the presence of metastatic RCC. The biopsy showed immunohistochemical positivity for CK AE1/AE3, PAX 8, and TFE3 (with a diffuse and strong positive pattern), while SALL4 and glypican 3 tested negative. The marked and widespread TFE3 staining strongly indicated the possibility of Xp11 translocation RCC.

**Figure 2:**
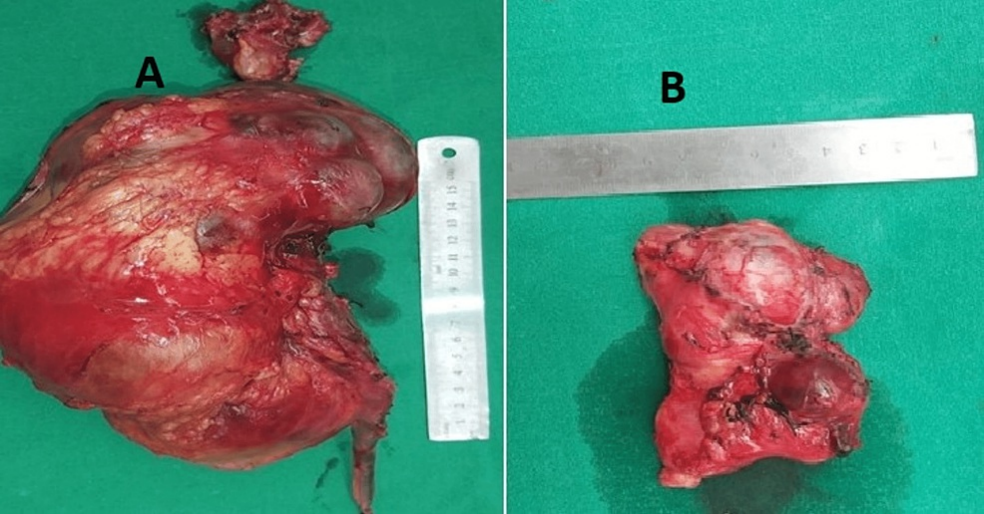
(A) Left renal tumor measuring 18 x 12 x 10 cm involving upper, middle, and lower pole of the kidney and left renal sinus fat. The left adrenal gland was also removed. (B) Left para-aortic node measuring 9.5 x 5 x 7 cm. Only one lymph node was involved, and extranodal extension was absent

On June 3rd, 2022, the patient underwent a left radical nephrectomy with retroperitoneal lymph node dissection after meeting the criteria for surgical treatment. During the surgery, the intraoperative findings revealed a large, predominantly solid with cystic components, renal mass measuring 18 x 12 x 10 cm (Figure 2). Additionally, there were enlarged retroperitoneal and para-aortic lymph nodes surrounding the renal vasculature and the aorta. The nodal mass was removed en bloc. Following the surgery, the patient had a smooth and uneventful recovery and was discharged from the hospital after five days. Upon macroscopic examination, the tumor displayed a varied appearance with extensive areas of necrosis.

**Figure 3:**
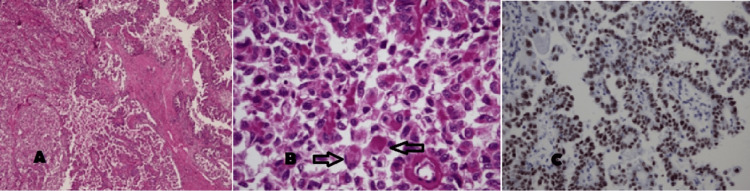
(A) Histological picture of the left renal mass seen in the radical nephrectomy specimen, composed of a mostly papillary (right) and focally solid (left) epithelial tumor with eosinophilic cells (H&E, 4X). High power view (B, H&E, 20X) showing a relatively solid area with eosinophilic to clear cells having globular eosinophilic inclusions in the cytoplasm (arrows) suggestive of Xp11 translocation RCC. TFE3 immunohistochemical stain (C) is strongly positive in the nuclei of tumor cells confirming the diagnosis

Microscopically, the tumor involved the renal sinus fat and hilar vessels (Figure 3). Its morphological features primarily consisted of a papillary pattern, with focal solid regions, and the presence of large eosinophilic to clear cells containing globular eosinophilic inclusions in the cytoplasm (Figure 3A, 3B) [6]. The tumor did not exhibit sarcomatoid or rhabdoid features; however, lymphovascular invasion was present. A conglomerated nodal mass (10 x 7 x 5.5 cm) involved with the tumor was removed, but no extranodal extension was observed. The immunohistochemical evaluation showed patchy positivity for CK7 and CKAE1/AE3, while TFE3 exhibited diffuse and strong positivity (Figure C). HMB45 and Melan-A were negative. The final diagnosis was Xp11 translocation RCC, which was confirmed through fluorescence in situ hybridization (FISH). The pathological stage was determined as T3aN1Mx, placing the patient at stage III according to the tumor, nodes, and metastases classification, eight edition.

Considering the aggressive course of Xp11 translocation RCC, the case was discussed in the multidisciplinary tumor board for adjuvant treatment consideration. However, due to the lack of evidence of benefit with these modalities on this unique pathologic entity and no other disease foci found on postoperative positron emission tomography-CT, adjuvant therapy was deferred to a possible future recurrence. The patient was advised to undergo regular chest, abdomen, and pelvis CT every three to six months for the first three years, and then 6-12 monthly for at least five years. Her six-month follow-up CT after surgery showed no signs of disease, leading to a decision to continue surveillance.

In March 2023, the patient developed clinical ascites, prompting a second CT with contrast for the chest, abdomen, and pelvis, which revealed left supraclavicular lymphadenopathy, peritoneal carcinomatosis, and ascites. A repeat biopsy confirmed the presence of the primary disease, leading to the recommendation of initiating systemic treatment with tyrosine kinase inhibitors (TKIs), specifically sunitinib, at an oral dose of 50 mg for two weeks followed by one week off. Although the possibility of combining TKIs with immunotherapy was discussed, it was deemed unfeasible due to financial constraints. As a result, she commenced treatment with TKIs alone. The plan involves conducting a repeat CT for the chest, abdomen, and pelvis in three months to assess the progression of the disease and make subsequent decisions regarding the course of action based on the assessment outcomes.

## Discussion

Xp11.2 RCC with TFE3 gene fusion is a rare tumor. Sidhar et al. were the first to describe this rare cancer [8], and the WHO recognized it as a distinct entity in 2004 [9]. Initially thought to be a tumor of the pediatric age group, it now comprises approximately 40% of pediatric RCCs and 1.6-4% of adult RCCs [10]. Prognosis is generally poor in adults compared to pediatric Xp11.2 RCC [11].

The present article aimed to report a rare case at our institute in Karachi, Pakistan, involving an 18-year-old lady with Xp11.2 RCC with TFE3 gene fusion of the left kidney and to review the relevant literature to better understand the characteristics of this rare type of cancer, as well as its treatment and prognosis.

Xp11.2 RCC is usually identified accidentally on imaging and typically presents as an asymptomatic, painless renal mass with local signs of gross hematuria, flank pain, or a palpable abdominal mass. Initial clinical data usually suggest an indolent clinical course despite the advanced stage. The common sites for metastases are the liver, lung, and retroperitoneal lymph nodes [11]. Patients with lymph node involvement at initial presentation are more frequent in children (58.8%) than in adults (28.8%). Surprisingly, it is associated with a poorer prognosis in the adult population, including shorter progression-free survival and overall survival compared to their pediatric counterpart [12].

However, recent reports have indicated that adults with Xp11.2 RCC have a more aggressive clinical course and poorer outcomes than conventional RCC types [11]. There are different gene fusions associated with Xp11.2 RCC, such as TFE3, CLTC-TFE3, PRCC-TFE3, and Nono-TFE3, with the chromosomal rearrangements t(X;17)(p11.2;q25), t(X;1)(p11.2;p34), t(X;17)(p11.2;q23), t(X;1)(p11.2;q21), and inv(X)(p11.2;q12), respectively [10]. The different gene fusions may be associated with different clinical and morphological characteristics [13]. The typical morphology of Xp11.2 RCC includes nested or papillary architecture, with cells having clear voluminous cytoplasm, eosinophilic hyaline globules, and psammomatous calcifications.

As these translocations are located on the X chromosome, it would appear reasonable to expect gender differences in this rare cancer; however, there is insufficient evidence to support this hypothesis.

The diagnosis of Xp11.2 RCC is based on microscopic appearance, TFE3 immunostaining, and genetic analyses. The most common diagnostic method of Xp11.2 RCC with TFE3 gene fusion is immunohistochemistry (IHC) assay using an antibody for the C-terminal portion of TFE3, as this cancer involves TFE3 protein overexpression. However, recent studies have reported that IHC for TFE3 is associated with a high rate of false-positive results [14]. Thus, the genetic identification of this rare cancer using FISH is an essential diagnostic method [14]. Our local study evaluated 18 cases suggestive of Xp11 translocation RCC, and only 50% of them showed strong nuclear TFE3 expression with a mean age of 24 years [15]. Because of the rarity of the disease and the unavailability of testing capabilities in our country earlier, including FISH testing for TFE3, most of the cases were not reported, but this has now changed since testing resources are now available [16].

About 20-40% of pediatric RCCs, mainly Xp11.2 translocation, affect children, while only 1-1.6% affect adults [12]. As the prevalence of RCC has increased, the incidence of Xp11.2 translocation is relatively more common in adults. Xp11.2 RCC normally presents at an advanced stage and with an aggressive clinical course and poor prognosis in adults [14]. The mean survival rate of up to two years in adults while up to 6.3 years in pediatric patients was reported by Srigley et al. Moreover, 15% of adult patients with Xp11.2 RCC have a history of previous chemotherapy use [12]. Hence, the possible role of previous chemotherapy as a causative etiology of Xp11 translocation RCC is to be confirmed [12].

The treatment for Xp11.2 RCC varies; however, there has been no established effective treatment to date. Xp11.2 RCC is treated similarly to conventional RCC. For localized Xp11.2 RCC with positive regional lymph nodes, surgery is the optimal treatment [17]. If the tumor is sized <7 cm, nephron-sparing surgery is considered a treatment option [18]. Various treatment options are available for patients with advanced and metastatic RCC, including immunotherapy, TKIs such as multikinase inhibitors including vascular endothelial growth factor, interleukin-2, and interferon-α, and the role of mammalian target of rapamycin inhibitors may be effective for Xp11.2 RCC [8]. Moreover, targeted agents, such as sunitinib, sorafenib, and everolimus, were applied [19]. Recent data showed the benefit of a combination of immunotherapy with multi-TKIs, including MET inhibitors, in non-clear cell metastatic RCC [12]. However, data regarding adjuvant treatment, including immunotherapy, is still under investigation, and no standardized adjuvant treatment has yet been established, despite its poor prognosis [12]. We still don't have enough. While undergoing sunitinib, the present strategy entails a review after three months utilizing scans to decide the next steps for further medical intervention.

## Conclusions

Xp11.2 RCC is often overlooked and requires a comprehensive approach for diagnosis using histomorphology, IHC, and FISH studies. In adult females, this rare subtype shows higher metastasis and a poor prognosis despite advancements in targeted and immunotherapy. Prospective studies on rare RCC subtypes, like Xp11.2 RCC, are underway. Radical nephrectomy remains the standard treatment for early-stage disease, while ongoing research focuses on advanced cases. Retrospective studies and case reports play a crucial role in tailoring treatments for similar patient conditions. By gathering more local data and collaborating internationally, we can develop targeted therapies and personalized approaches to improve overall management and outcomes for patients with Xp11.2 RCC.
